# The Effects of Eight Weeks Selected Aerobic Exercises on Sleep Quality of Middle-Aged Non-Athlete Females

**DOI:** 10.5812/ircmj.16408

**Published:** 2014-07-05

**Authors:** Zahra Kashefi, Bahman Mirzaei, Ramin Shabani

**Affiliations:** 1 Department of Physical Education, Islamic Azad University, Rasht Branch, Rasht, IR Iran; 2 Faculty of Physical Education, University of Guilan, Rasht, IR Iran

**Keywords:** Aerobic Exercise, Middle-aged, Sleep Disorders

## Abstract

**Background:**

Sleep is considered as one of the most important factors, directly influencing mental and physical health components. In the last decade, low sleep quality - i.e. poor sleep - has become one of the major problems of the individuals, especially in middle-aged women. Low quality sleep also directly influences memory, functional components, nutrition, and mood.

**Objectives:**

This study aims to detect the effect of selected aerobic exercises on sleep quality in non-athlete middle-aged women.

**Materials and Methods:**

Fifteen non-athlete middle-aged women participated in this study, all of them suffered from insomnia. Pittsburgh questionnaire was used for determining sleep quality in this sample. Four indices including sleep duration, sleep disturbance, sleep latency, and sleep efficiency have been investigated through this. The period of exercises included eight weeks, three one-hour sessions each week. The sample group was trained during eight weeks through performance of selected aerobic exercises including three groups: sequential movements equip mental movement and movements on the pad. The selected protocol included performance of exercises: 10 minutes for warm up, 10 minutes for sequential movements, 20 minutes for movements by using equipment, 15 minutes for movements performed on the pad, and 5 minutes for cooling down. The exercises during the first four weeks have been presented with 60% increase of the heart rate, and 75% increase during the second four weeks. The sample group was provided with Pittsburgh questionnaire at the beginning of the exercises and the end of each week. The information of each person was registered.

**Results:**

The results showed that the mean of sleep duration, sleep disturbance, sleep latency, and sleep efficiency indices significantly reduced 32%, 22%, 30%, 14% and 36%, respectively. The results also showed that the trend of changes in sleep duration, sleep disturbance, sleep latency, and sleep efficiency indices had significant descending trend.

**Conclusions:**

We concluded that eight weeks of aerobic exercises can significantly increase sleep quality in middle-aged women.

## 1. Background

Nowadays, industrialized lifestyle has influenced the components of individuals' health by direct effects of the technology-related factors. Most of the health-related factors have undergone dramatic changes, which have negatively affected man's health. Sleep is considered as one of the most important factors, directly influencing mental and physical health components. In the last decade, low sleep quality - i.e. poor sleep - has become one of the major problems of the individuals, especially in middle-aged women. Low quality sleep also directly influences memory, functional components, nutrition, and mood (1). This problem has been prevailed among middle-aged people regarding their age. Based on physical and mental components, it increases the risk of incidence of numerous diseases in people. Poor sleep is considered as one of the important factors, which changes hormone balance and aggravates the hormonal changes in mature women entering upon middle-age. According to the evidence, these hormonal disorders significantly influence different body organs including heart, brain, and action systems (2).

The researches have shown that more than 50% of middle-aged women suffer from low sleep quality or insomnia due to different causes; noted by females, especially middle-aged perimenopause women. Researches have demonstrated that 20% of perimenopause women sleep less than six hours per day (3). Longitudinal researches, conducted on more than 10,000 people during 17 years have shown that the risk of death increases 1.7 fold in patients with decreased sleep duration from eight hours to five hours or less, especially in middle-aged individuals, compared to those with normal sleeps (4). Another research, conducted in the United States, also showed that 74% of adults suffer from insomnia and spend most nights with low sleep quality. In addition, 39% of people sleep less than seven hours during day and night (5).

According to the mentioned issues, the effects of low sleep quality influence two components: physical and mental factors. Sleep quality directly affects physical activities, especially voluntary and involuntary responses. In competent individuals with low quality sleep, the level of special daily activities has significantly declined. In addition, potential daily affairs response capabilities have been declined in some cases (6).

Three major groups of physical activities including cardiovascular, nervous, and hormonal systems are directly influenced by sleep quality. (7). Researches have shown that inadequate sleep or related problems, increase the risk of cardiovascular diseases and are also considered as effective factors in heart failure (8). This issue, leads to the irregulation of involuntary functions within a specified period. For example, insufficient night sleep may result in arrhythmia and irregular heartbeat. Furthermore, it may lead to sudden hypertension, which increases the risk of myocardial infarction (9).

Different researches have been conducted on sleep quality and brain activities; most of them supporting the relationship between the low quality sleep and the reduction of memory and other brain functions (10). As the categorization of information in brain is mostly done during sleep, high quality and adequate sleep directly influences retrieval of stratified stored memory. Brain is the center for directing and issuing commands for all organs to perform involuntary and voluntary activities. This center performs analyses and retrieval of the required capabilities during sleep, therefore sleep quality is of high importance for improvement and regulation of different commands issuing (11). Low sleep quality or poor sleeps directly influence individuals' mental and psychological components, their physical and behavioral components, and their moods. (12).Hormonal producing mechanisms and the level of hormones secretion are considered as another factor, which can directly be affected by sleep quality component. Hormones are responsible for a major part of the regulation of different body activities and also for behavioral balance. Several researches have been conducted on the relationship between sleep quality and hormones secretion. hypophysis, a gland located in brain, establishes a relationship between sleep quality and balance of the hormones (13). Feminine hormones, likewise other hormones related to general system, are influenced by sleep quality. Specific researches have shown that estrogen and progesterone, especially in middle-aged women, undergo some changes during night sleep, which influence the level of the hormones during the day (14). In addition to the mentioned hormones, cortisol, having wide physiological effects in body, is directly influenced by the quality of night sleep. This hormone has an important role in proteins' decomposition and glucose, fructose and lactose neogenesis, and also in body defense against different risks such as infections, quality of night sleep also greatly influences the level and conditions of individuals' nutrition, digestion factors and food intake substances (15).

Researchers have shown that the quality of night sleeps affects factors anxiety and depressions factors. Individual with insomnia or long-lasting low quality sleep significantly increases anxiety components (16). 

Several studies have been performed regarding the effects of exercises, especially aerobic exercises, on reduction of the risk of heart attacks and also the regulation of blood pressure. These studies have shown that performance of moderate aerobic exercises results in a calm and strong body, leading to better body organs' performance (17). The effects of performing regular exercises on the regulation and balance of different hormones secretion and their absorption are also very crucial. It should be mentioned that the hormones' balance is considered as one of the most important factors in health maintenance and elimination and alleviation of body organs' disorders (18). Aerobic exercises, improves body's functional and behavioral patterns, and in addition to removing the sudden pressures on different systems of body, these exercises have significant effects on systems compatibilities with exercises (19).

This discussion, partly, shows the effect of sleep duration and its quality on health components; highlighting the importance of sleep. Today, due to side effects of medications, physicians and physiologists prefer not to prescribe medicines and they recommend patients to use alternative therapies (20). Regular exercises are among the alternatives prescribed for improving sleep quality. It is evident that improving sleep quality through performing exercises not only helps improving the health of body's different mechanisms, but also it is useful in regulating cardiovascular system, brain function and mental and psychological state e.g. anxiety and mood. 

## 2. Objectives

This research aims to investigate the possibility of substitution of narcotic drugs by some exercises under the title of aerobic exercises.

## 3. Materials and Methods

### 3.1. Definitions

#### 3.1.1. Sleep Duration

Sleep duration is the total of continuous hours of sleep during one night (for example from 8 pm to 8 am). Short sleeps are those sleeps, which last less than 5 hours and long sleeps are those, which last more than 8 hours (21).

#### 3.1.2. Sleep Disturbance

Sleep disturbance includes difficult sleep initiation, sudden waking up from long and short sleeps repeatedly and not having profound sleep (22).

#### 3.1.3. Sleep Latency

Sleep latency is the inability or spending long time to fall asleep (usually it takes 15 minutes). Any activity, after lying down in bed, which causes sleeplessness, is called sleep latency (23).

#### 3.1.4. Sleep Efficiency

Sleep efficiency is calculated as a proportion of the time in profound sleep by the time lying in bed. In other words, sleep efficiency includes the ratio of real sleep from the duration of person lying in the bed (24).

### 3.2. Sample

The sample includes 15 middle-aged women, in the age between 30and 50 years. These samples have not been among professional athletes and their ability of activities was in a normal level, and had no physical problems. They were informed of the benefits and potential injuries rising from these exercises; according to the Sports Research Standards, all persons who participated in the research provided written consent form.

### 3.3. Selected Aerobic Protocol

The selected protocol that used in this study referred from standards of fitness organizations in order to provide the sample group with Aerobic exercises (25). These standards include six physical fitness's factors: power, muscular strength, muscular endurance, flexibility, coordination and agility. The five components in sequential movements and usually rhythmic movements were used through a specific program under the title of selected protocol in this research. The selected protocol included two kinds of exercises: general exercises and special exercises (26, 27).

The program of general exercises included movements for warming up and cooling down in order to prevent the occurrence of potential injuries. The most important exercises in warm up stage included jacking, dynamic and static movements, regular movements from top to down of the body, accompanying with light exercises. The most important general exercises in cooling down stage included stretch movements, preferentially static movements, in order to return body to the first state. The period for general exercises of warm up in the beginning of the exercises lasted 10 minutes in selected protocol (28, 29). The program of special exercises was consisted of main aerobic movements including sequential movements, equip mental movements, and movements on the pad which were performed after warm up stage. The mentioned exercises were performed with 60% intensity of maximum heart rate (HR) for the first four weeks and 75% intensity of maximum HR for the second four weeks. The following formula was used for calculation of maximum HR: Maximum HR = Age-220. The special exercises included three groups of movements: sequential for 10 minutes, equip mental for 20 minutes and movement on the pad, which lasted 15 minutes, respectively (25).

### 3.4. Sequential Movements

The most important movements among sequential movements included March (in, out) Step touch, V-step, Easy walk, Box step, Knee up, Forward mambo, Baby mambo, Jazz square, Grape vine, Kick and Chasse mambo. These movements have been performed in combined and sequential forms, generally rhythmic and accompanying with music within the first 10 minutes of the special exercises (26).

### 3.5. Equip Mental Movement

The equipment being used for performance of these exercises included light dumbbell, gym stick, step, and gym ball, as well as the wall of the gym. The important movements related to dumbbell included dumbbell curl, dumbbell triceps, lateral raise, front raise, chest dumbbell, dumbbell bench press, dumbbell crutch, dumbbell squat. Important movements related to gym stick included the quadriceps with gym stick, thigh outside gym stick, side by side with gym stick, abdominal with gym stick. The most important movement by using gym ball included sit up on the ball, pelvis lifting, the sides stretch on the ball, and the most important movements by using wall included chest press, quadriceps contraction, hamstring stretch, and abductor and adductor stretch, raise stretch, body twisting stretch. According to a specific program, the above mentioned movements were performed within the second 20 minutes of the special exercises. Table 1 shows the program of these movements for 8 weeks.

**Table 1.** Equip Mental Movements Program During Each Week

Session During Each Week	Movements
**First week**	The movements related to dumbbell and gym ball
**Second week**	The movements related to the step and the wall
**Third week**	The movements related to gym stick and the wall

### 3.6. Movements on the Pad

The most important of the movements included half sit up, crunch, reverse crunch, abdominal movement, side stroke, lunge, abdominal oblique and bridging. These movements have been performed in the last 15 minutes of each session for special exercises. The movements related to cooling down stage have been performed after the end of 45 minutes performance of special movements. This stage was designed for returning the heart rate to the initial situation during the last 5 minutes of each session. The important movements included static stretch movements and relaxation (26). The Table 2 shows the general and special movement under title “Selected Aerobic Protocol” used in this research (30).

**Table 2.** The Selected Aerobic protocol

Type	Important Movements	Time
**General**		
Warm up	dynamic stretch movements, moderate intensity exercises, jacking	10
Cooling down	Static stretch movements, relaxation movement	5
**Special**		
Sequential movements	march, v-step, easy walk, mambo, step touch, grape vine	10
Equip mental movements	dumbbell curl, dumbbell triceps, crutch dumbbell, quadriceps with gym stick, sit up with ball, chest press on the wall, dumbbell scout	20
Movements on the pad	half sit up, full sit up, lunge, oblique coral, crunch	15

### 3.7. Determination of Sleep Quality

Pittsburgh questionnaire is an efficient method for measuring sleep quality. This questionnaire has been standardized during the last decades and it is usually used in many recent researches, which are performed about quality of sleep. Pittsburg questionnaire included four groups of questions, usually completing during one month by sample group. The first group of questions included the time of falling asleep and the time of waking up. The second group was about the issues caused inadequate sleep in sample group. The third group included the questions about the psychological and mental reasons for inadequate proper sleep. The fourth group included questions answered by another person (partner or someone who knows the condition of the person's sleep) (31). Pittsburgh questionnaire included eleven questions, the fifth question was consisted of nine parts and the eleventh question was consisted of five parts. The latest researches have shown that the validity and reliability of this questionnaire is 83% (32). All questions in this questionnaire have been translated into Persian and some parts of the questionnaire have been modified to be proper for local use (domestic questionnaire). During the 8-week selected aerobic exercises, people have been provided with a questionnaire in the beginning of each week and the copies, completed by them, were returned at the end of each week. Therefore, we have eight completed questionnaires for each person. Pittsburgh sleep quality indices include one general index and four subscale of sleep quality (duration of sleep, sleep disturbance, sleep latency, and sleep efficiency) (33). The general index of sleep quality includes the four a bow indices and presents a general view of a person’s sleep. The range of these numerical indices is 0 to 21. The greater numbers show low quality of sleep and the smaller numbers show the good and high quality of sleep. The number “five” is usually considered as a limit for low quality of sleep and good quality as a cut point. In other words, the numbers which are greater than this limit 5 indicate low quality of sleep, and the smaller numbers indicate the high quality of sleep for a person. The Pittsburgh general index is a combination of four indices (duration of sleep, sleep disturbance, sleep latency, and sleep efficiency). These sub scale of sleep quality are within the range of 0 to 3.

### 3.8. Statistical Analysis

Table 3 shows the descriptive statistics, means and standard deviation for anthropometric factors of the samples. The numbers in parenthesis indicate the standard deviations. The following table shows the means for sleep quality indices before and after the performance of 8-week aerobic exercises. The numbers in parenthesis indicate the standard deviations. The digits in column before and after show the reduction in the levels of sleep quality indices. In the following analyses, we consider on the main question: These reductions are statistically significant ? (Table 4)

**Table 3.** Anthropometric Characteristics of the Sample Group^a^

Variables	Means ± SD
**Height, cm**	161.95 ± 5.24
**Weight, kg**	68.23 ± 10.75
**Waist, cm**	84.79 ± 11.02
**Hip, cm**	104.11 ± 9.79
**BMI**	26.06 ± 4.28
**WHR**	0.81 ± 0.62

^a^Abbreviations: BMI, body mass index; WHR, waist–hip ratio

**Table 4.** The of of Means and Standard Deviation Related to Sleep Quality Indexes

Indexes	Before Exercises	After Exercises
**Duration of sleep**	2.12 ± 0.61	1.43 ± 0.75
**Sleep disturbance**	2.74 ± 0.18	2.11 ± 0.22
**Sleep latency**	2.11 ± 0.33	1.47 ± 0.69
**Sleep efficiency**	2.26 ± 0.43	1.94 ± 0.68
**Sleep quality**	11.25 ± 2.12	7.16 ± 3.18

**Figure 1.**
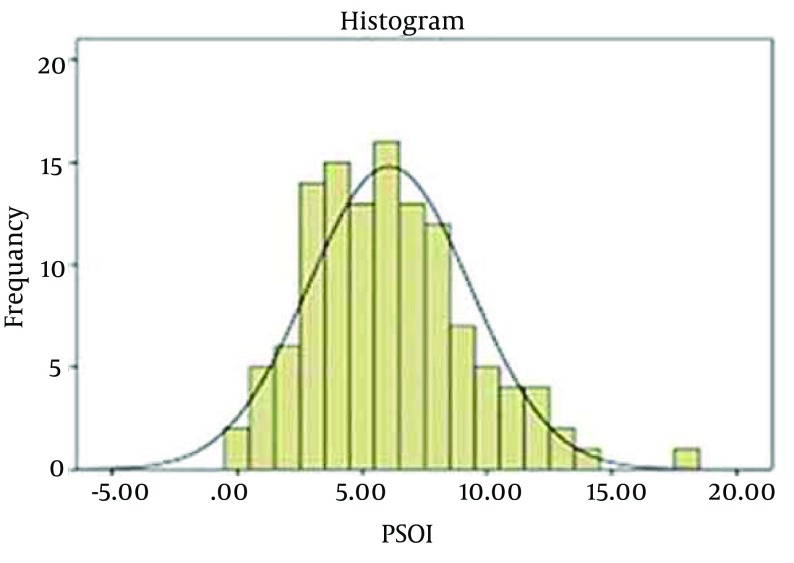
Frequency Histogram of General Sleep Quality

The main statistical analysis for detecting of effect of eight weeks of aerobic exercises on sleep quality is regression analysis. This analysis can obtain the trend of changes in means of the sleep quality indices in the period. First, we test the normality of the indices by Kolmogorov-Smirnov test. If the test improves the normality of these indices, we can select the parametric regression methods for investigations. Figure 1 presents the frequency histogram of general sleep quality index during eight weeks performance of exercises. According to the diagram, it seems that this index has the normal distribution.

**Figure 2.**
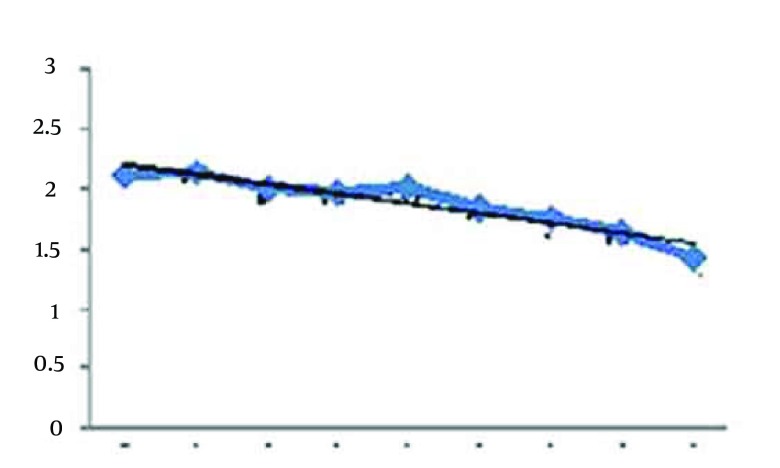
Trend of Sleep Duration Index During the 8 Weeks

Table 5 shows that the hypothesis normality of distribution of sleep quality indices are not rejected (all P > 0.05). Thus, the parametric analysis approaches can be used for investigation of indices variations occurred due to the eight week Aerobic exercises. The Figure 2 shows the means for sleep duration index for sample group during eight weeks.

**Table 5.** The Result of Kolmogorov-Smirnov Test for Sleep Quality Indices

	Absolute Value	Positive	Negative	Z	Sig
**General quality**	0.102	0.102	-0.112	0.117	0.365
**Sleep duration**	0.179	0.179	-.0181	1.093	0.221
**Sleep disturbance**	0.262	0.262	-0.240	1.486	0.282
**Sleep latency**	0.130	0.130	-0.151	1.242	0.256
**Sleep efficiency**	0.257	0.257	-0.215	1.376	0.162

**Figure 3.**
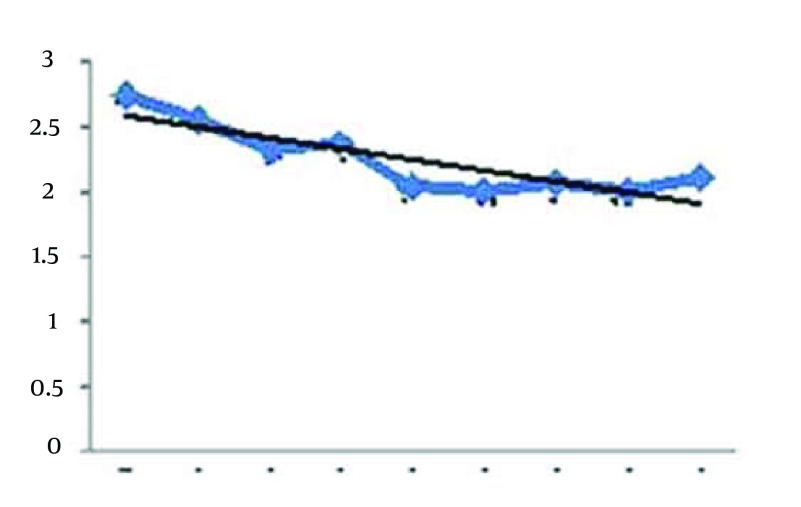
Trend of Sleep Disturbance Index During the 8 Weeks

Considering Figure 2, it seems that the sleep duration index has been decreased with the continuation of the exercise sessions. The Pearson correlation test between sleep duration index and exercises sessions is r = 0-0.224, P = 0.021. Therefore, significant correlation was seen between sleep duration and continuing aerobic exercises. The results of regression analysis between these two variables are presented in Result section. Figure 3 shows the trend of sleep disturbance during the 8 weeks. It seems the decreasing trend between the disturbance index and the weeks of Aerobic exercises. The Pearson correlation between sleep disturbance index and exercises sessions is r = 0 - 0.308, P = 0.003.

**Figure 4.**
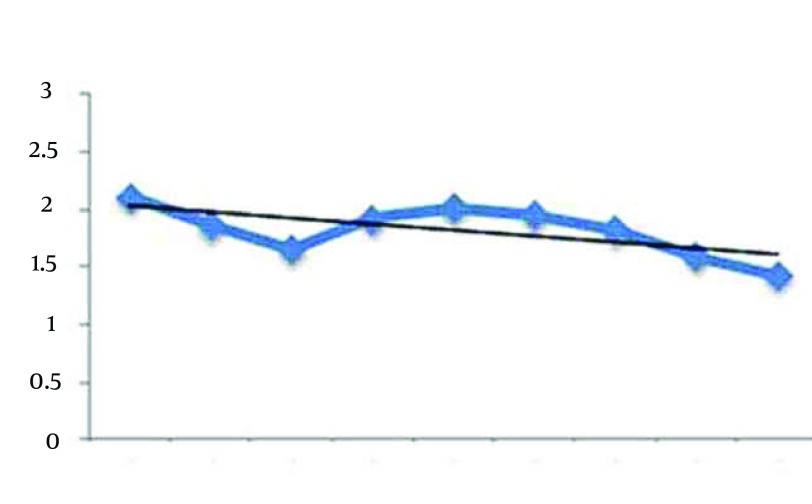
Trend of Sleep Latency Index During the 8 Weeks

**Figure 5.**
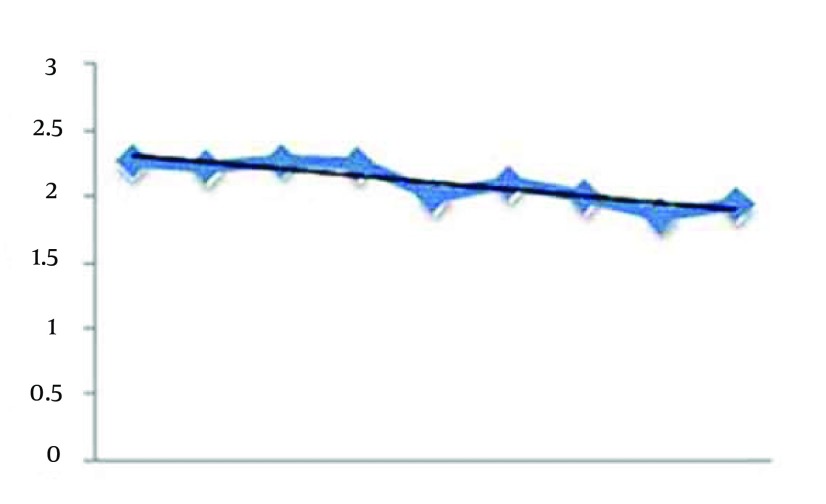
Trend of Sleep Efficiency Index During the 8 Weeks

Figure 4 presents the sleep latency index during the eight weeks aerobic exercises. This shows shows that by increasing the weeks of exercises the sleep latency index is decreased. The Pearson correlation between sleep duration index and exercises sessions is r = -0.206, P = 0.025. Significant correlation was seen between weeks of aerobic exercises and sleep latency index. Figure 5 shows the trend of sleep efficiency index during the eight weeks. According to this figure, it seems that sleep efficiency is approximately constant in eight weeks. In other words, we have not tangible changes in this index by increasing the weeks of exercises. The Pearson correlation between sleep efficiency index and exercises sessions is r = -0.089, P = 0.117.

**Figure 6.**
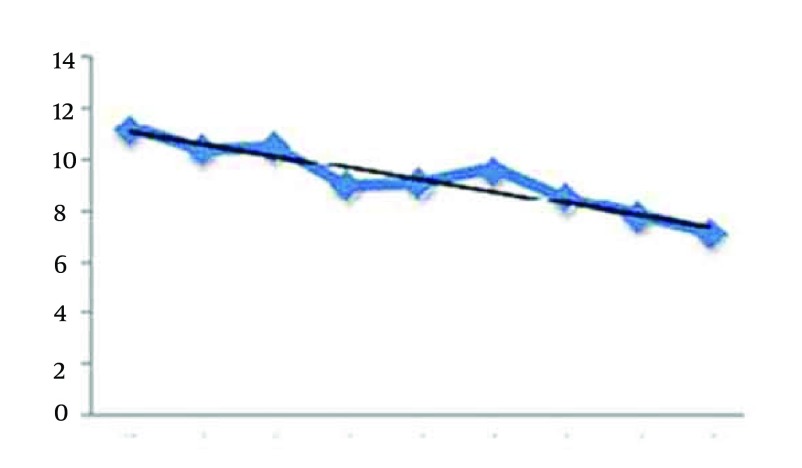
Trend of General Sleep Quality Index During the 8 Weeks

Finally, Figure 6 presents the trend of general sleep quality index during the eight weeks of aerobic exercises.

The shows shows a negative relation between general sleep quality and the weeks of exercises. Therefore, by increasing the weeks of exercises, the general sleep quality index decreases. It means that continuing the aerobic exercises is useful for improve quality of sleep (r = -0.376, P = 0.000).

## 4. Results

Regression analysis is the main analyses for investigation of relation between sleep quality indices and aerobic exercises. The main results of this analysis for each index are as follow:

1. Sleep duration: Regarding to the F of linear regression between sleep duration index as dependent variable and the weeks of exercises as independent variable (F = 58.86), significant relation was seen between variables (P = 0.000). In addition, the slope of the line is -0.082 (P = 0.024). Therefore, aerobic exercises have significant effect on sleep duration. Then, by increasing the weeks of exercises, sleep duration decreases.
2. Sleep disturbance: The quantity of F related to linear regression between sleep disturbance index (dependent variable) and weeks of aerobic exercises (independent variable) is 9.49 (P = 0.003). The slope of the line is -0.084 (P = 0.025). These results show that we have a negative significant relation between sleep disturbance index and weeks of aerobic exercises.
3. Sleep latency: The F related to the linear regression between sleep latency as dependent variable and weeks of aerobic exercises as independent variable is 7.57 (P = 0.020). The slope of this line is -0.101 (P = 0.000). Therefore, the relation between the two variables is significant. It means that by increasing the weeks of exercises, sleep latency index decreases.
4. Sleep efficiency: Regarding to the linear regression between sleep efficiency index as dependent variable and weeks of exercises as independent variable, the F is 0.87 (P = 0.253). The slope of the line is -0.051 (P = 0.154). The results presented that there is no significant linear relation between the sleep efficiency index and weeks of exercises.
5. General sleep quality: the linear regression analysis between general sleep quality index (dependent variable) and weeks of aerobic exercises demonstrated an F of 15.588 (P = 0.000). The slope of this line is -7.714 (P = 0.000). Therefore, significant negative relation was seen between general sleep quality index and weeks of exercises. It means that general sleep quality index significantly decreases by increasing the weeks the exercises. Therefore we can conclude that aerobic exercises is a most powerful method for ameliorating sleep quality.

## 5. Discussion

During the last decades, widespread researches have been conducted regarding the effect of different factors on sleep quality. Some of them studied the effects of neurotoxin drugs or sedative drugs on sleep quality variations. However, the side effects of the mentioned drugs have made researchers to consider non-pharmaceutical effects on the sleep quality variations. In this respect, some researches and investigations have been widely performed studies about the effects of exercise, as a non-pharmaceutical approach on sleep quality. Most of these researches indicate the positive effects of exercises on sleep quality (34). The current findings also support the efficiency of a set of exercises (aerobic exercises) on sleep quality. According to the investigations results, performance of eight week selected aerobic exercises by a group of middle-aged women is an effective on improving sleep quality indices. In other words, these exercises could reduce the indices related to sleep quality in Pittsburgh questionnaire such as indices of sleep duration, sleep disturbance, sleep latency, sleep efficiency and of course Pittsburgh sleep questionnaire index (PSQI). It should be mentioned that the each PSQI index range between 0 to 3, as the smaller numbers indicate better sleep quality and vice-versa. Thus, reduction of these indices indicates the improvement of sleep quality. In the following, we present some researches, performed about investigation of exercises effects on sleep quality. Moreover, we present the researches that are consistent with finding of our research.

Lira et al. indicated that regular moderate Aerobic exercises reduce significantly sleep quality indices; the results of this investigation are consistent with the results of our current research. As different blood parameters, including triglyceride level, are considered as metabolism components, the mentioned exercises have positive effects on the balance of these parameters as well. Aerobic exercises have also been effective for balancing hormonal level, and for reduction of LDL cholesterol and triglycerides. As the above-mentioned issues have paved the way for different diseases including diabetes, obesity, and metabolic syndromes, these exercises have been very useful and effective for improvement of physical conditions and health of individuals. Perhaps these results are due to the compatibility of skeletal muscles with the mentioned exercises, leading to the reduction of triglyceride level in cells. The general result shows that hormonal balance is effective in improvement of those antioxidants pertaining to skeletal muscles and to tranquilizing the muscles and this balance can indirectly result in the reduction of sleep latency index. It should be mentioned that it is true about sleep efficiency index as well (35).

Another example is Sengul et al. They designed a study aiming to investigate the effect of various exercises on sleep quality components. The results showed that sleep quality indices had been changed significantly at the end of exercises period (12 weeks), and the quality of sleeps in individuals had been increased significantly. The researchers considered the increasing of metabolism and elimination of mental tensions and anxiety, arising from over-concentration of energy, as the main reasons for positive effects of exercises on the improvement of sleep quality. As increasing metabolism can increase blood pressure in muscles, the muscle cramps can occur easier due to the performance of exercises, and it makes a mental state leading to the reduction of indices related to sleep disturbance (36). Pasous et al. studied the effects of moderate aerobic exercises on sleep quality and cardiovascular performance. The aim was to investigate the effect of exercises time of morning and afternoon performing exercises on sleep quality indices. The findings showed that the time of performing the exercises had not been affected the sleep quality, but generally, these exercises led to the improvement of two indices: sleep latency and sleep efficiency. This investigation is consistent with our research (37). Also in the research, a strong relationship has been seen between mental states of the individuals and their sleep component. Thus, improvement of the psychological and mental state of individuals directly affects sleep quality, especially their sleep latency index. Based on the results of this research, as aerobic exercises are accompanied with some coordinated and rhythmic movements, serotonin and dopamine hormones are balanced, affecting directly the person's vividness and liveliness. It was also shown that the increase in the mentioned hormones improves the sleep component such as sleep general quality index (38).

King et al. showed that long term, moderate exercises could improve sleep quality indices The results presented the mentioned exercises improve as the process of the activities in sleep second stage, the stage in which brain waves variations occur, and it causes the reduction of sleep latency and also it produces feeling of calmness. This happens in sleep second stage because brain waves become smooth; thus, this smoothness reduces sleep latency index and also improves of general index of sleep quality by a great amount (39). This finding is also consistent with results of our research.

Another example of this subject is a research designed by Pepard et al. about middle-aged women with insomnia. It was showed that exercises could lead to the reduction of sleep disturbance. As this reduction is effective for improving sleep efficiency index, these exercises generally, result in the improvement of sleep quality. It should be noted that this research is consistent with our research. This study showed that sleep quality indices are sensitive to performance of regular exercises, especially planned exercises under a specific protocol. This reason has been explained in this research so that in case that these movements are performed rhythmically, they shall result in an increase in endorphin hormone, and makes the person feel liveliness during a day. This effectively helps the reduction of negative thoughts before sleep, and therefore improves sleep latency index and reduces sleep disturbance index significantly (40).

Pang Lee et al. have investigated the effect of exercises on sleep quality. In their research, the sample group was divided into four subgroups: underweight, natural weight, overweight, and obese individuals. The effects of performance of exercises on sleep quality indices were investigated by Pittsburgh questionnaire during six months. The results showed that for three subgroups with natural weight, overweight, and obese individuals, Pittsburgh sleep quality indices improved due to performance of regular exercises. However, exercises had not significance effect on sleep quality in the underweight subgroup, This results are not consistent with our research. The reason for why sleep quality has not been affected by exercises in the underweight subgroup is not mentioned. The level of calorie intake in the mentioned group is generally less than the required level, therefore the performance of exercises results in the reduction of calorie intake level and loss of the required energy accordingly. This is considered as a major factor for increasing sleep disturbance or non-variation in sleep quality in the sample group (41).

Tatum et al. investigated the relationship between physical activities and sleep quality. They divided physical activities into three types: low, moderate, and heavy activities. The results show that performance of low physical exercises has little effect on sleep quality variations. However, performance of heavy and moderate exercises has improved sleep quality indices. It seems that this result is not consistent with our findings, because the effect of low exercises activity on sleep quality was intangible. But upon perusing this research, we found that the exercises in which heart rate has not been increased in comparison with heart rate in rest, i.e. low exercises, could not affect sleep quality. This is because metabolism variations occur upon tangible increase of heart rate due to performance of physical exercises. As metabolism variations affect sleep quality indices, these exercises cannot affect the improvement of the sleep general quality. It should be noted that most aerobic exercises are considered as heavy and moderate exercises; therefore, they can improve sleep quality (42).

### 5.1. Practical Suggestions

1. Developing the research by including some psychological components such as depression and anxiety factors.
2. Expand the research by fixing the diet’s woman for the sample in training period.
